# Mounting Angle Prediction for Automotive Radar Using Complex-Valued Convolutional Neural Network

**DOI:** 10.3390/s25020353

**Published:** 2025-01-09

**Authors:** Sunghoon Moon, Younglok Kim

**Affiliations:** Department of Electronic Engineering, Sogang University, Seoul 04107, Republic of Korea; shmoon@sogang.ac.kr

**Keywords:** automotive radar system, complex value, convolutional neural network, mounting angle, deep learning

## Abstract

In advanced driver-assistance systems (ADASs), the misalignment of the mounting angle of the automotive radar significantly affects the accuracy of object detection and tracking, impacting system safety and performance. This paper introduces the Automotive Radar Alignment Detection Network (AutoRAD-Net), a novel model that leverages complex-valued convolutional neural network (CV-CNN) to address azimuth misalignment challenges in automotive radars. By utilizing complex-valued inputs, AutoRAD-Net effectively learns the physical properties of the radar data, enabling precise azimuth alignment. The model was trained and validated using mounting angle offsets ranging from −3° to +3° and exhibited errors no greater than 0.15° across all tested offsets. Moreover, it demonstrated reliable predictions even for unseen offsets, such as −1.7°, showcasing its generalization capability. The predicted offsets can then be used for physical radar alignment or integrated into compensation algorithms to enhance data interpretation accuracy in ADAS applications. This paper presents AutoRAD-Net as a practical solution for azimuth alignment, advancing radar reliability and performance in autonomous driving systems.

## 1. Introduction

Advancements in advanced driver-assistance systems (ADASs) have increasingly highlighted the critical role of various sensors in recognizing and interpreting the environment surrounding a vehicle [1,2,3]. An ADAS utilizes sensors such as radar, cameras, and lidar, either independently or through complementary fusion technologies, to support functionalities that enhance vehicle safety and automation [2,4]. Camera sensors are cost-effective and capable of recognizing and classifying objects using image data. However, they are sensitive to environmental conditions such as strong light, shadows, and fog and do not provide direct measurements of distance and velocity [3,4,5]. On the other hand, lidar provides high-resolution distance and velocity measurements, enabling precise object classification. However, its short wavelengths make it sensitive to atmospheric absorption and weather conditions, which limit its capability for long-range measurements. Additionally, its high cost poses a significant disadvantage [4,5,6]. By contrast, radar serves as a critical component in ADASs because of its cost-effectiveness, robustness to adverse weather conditions, and capability for long-range measurements, despite its comparatively lower resolution [4,5,6].

However, changes in the mounting angle of an automotive radar, caused by external forces such as impacts or vibrations, can severely affect object detection and tracking, degrading the overall system performance. In particular, minor angular misalignments in forward-mounted long-range radars can lead to significant distortions in long-range target location information. As a result, these misalignments can compromise the safety and operational reliability of ADASs [7]. Therefore, accurately estimating mounting angle offsets is crucial to maintaining the integrity of radar-based measurements and ensuring reliable system performance.

The estimation process of a vehicle’s mounting angle involves two primary methods. The first method entails detecting the mounting angle offset within single-frame data. The second method focuses on estimating a stable mounting angle by employing filtering techniques and statistical methods across multi-frame data. Existing studies on the first method have predominantly concentrated on detecting elevation mounting angle offsets in automotive radar systems [7,8,9]. However, these studies are limited to classification tasks, resulting in mounting angle offset predictions with limited resolution. To achieve greater accuracy, further exploration of regression-based approaches is necessary. Furthermore, due to the primary focus on elevation mounting angle offsets, this approach is fundamentally unsuitable for accurately estimating azimuth mounting angle offsets. Meanwhile, the second method uses conventional methods to detect mounting angle offsets in each frame. These are followed by post-processing methods, such as statistical techniques or Kalman filtering, to reduce noise and estimate stable offsets across multiple frames [10,11,12,13,14]. Nevertheless, these studies have predominantly focused on post-processing methods for multi-frame data and have not emphasized the need for accurate mounting angle offset detection at the single-frame level.

This study introduces a novel model, the Automotive Radar Alignment Detection Network (AutoRAD-Net), which is designed to precisely estimate single-frame mounting angles and optimize the azimuth alignment of automotive radar. The proposed approach addresses existing research gaps by overcoming the limitations of previous studies that primarily emphasized elevation-angle detection and relied on classification models, which are inherently less accurate than regression models. Furthermore, AutoRAD-Net mitigates the shortcomings of existing post-processing methods by providing a comprehensive solution for accurate and reliable azimuth alignment. The proposed model leverages a complex-valued convolutional neural network (CV-CNN), a neural network architecture designed to process both phase and magnitude information in the complex domain, leveraging the extensive information embedded in radar data for an enhanced representation. A CV-CNN enables more sophisticated learning and prediction than that provided by traditional real-valued models [15,16,17,18,19]. AutoRAD-Net effectively learns the physical characteristics of radar data by utilizing complex-valued inputs, overcoming the limitations of existing approaches and achieving high accuracy in angle estimation. This approach combines the physical properties of automotive radar data with the advantages of complex-valued representation, effectively leveraging critical information underexplored by conventional real-valued models. Furthermore, this study validates the performance of the AutoRAD-Net model using real-world measurement data and presents the model as a practical solution to the azimuth alignment challenge in automotive radar systems.

The remainder of this paper is organized as follows. Section 2 describes the measurement methods, data preprocessing procedures, and the proposed AutoRAD-Net model for mounting angle offset estimation. Section 3 presents the results of the AutoRAD-Net model, compares them with those of conventional methods, and discusses the performance differences and their implications. Finally, Section 4 concludes the paper.

## 2. Materials and Methods

### 2.1. Measurements

#### 2.1.1. Configuration of Vehicle and Radar System

**Figure 1:**
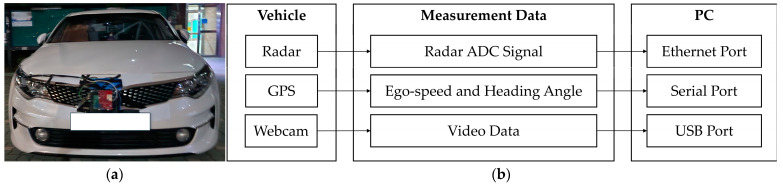
Measurement setup: (**a**) vehicle equipped with radar, Global Positioning System (GPS), and webcam; (**b**) data flow from radar, GPS, and webcam to the laptop.

The measurement setup used in this study included an AWR2944 board from Texas Instruments (TI) and an mmWave sensor designed for automotive radar applications operating within the 76–81 GHz frequency range [20]. To acquire analog-to-digital conversion (ADC) samples, the AWR2944 board was connected to a TI DCA1000 module for raw radar data acquisition [21]. The *ego speed* and *heading angle* were measured using a Global Positioning System (GPS) module to characterize the state of the vehicle in relation to the collected data. Driving conditions, including environmental and road factors, were recorded using a webcam for contextual monitoring during data collection. The sensors, including radar, GPS, and a webcam, were integrated into the vehicle used for data collection. A laptop was used to control the sensors and to acquire and store the collected data. Figure 1 illustrates the measurement setup, including the vehicle and radar system configurations.

#### 2.1.2. Waveform Design of DDMA-Based MIMO Radar

In this study, a Doppler-division multiple-access (DDMA)-based multiple-input, multiple-output (MIMO) waveform configuration was implemented in the automotive radar system [22,23,24].

**Figure 2:**
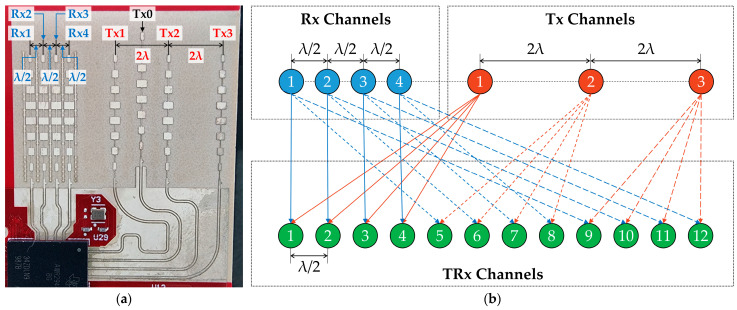
Configuration of Doppler-division multiple-access (DDMA)-based multiple-input, multiple-output (MIMO) radar system: (**a**) antenna layout-illustrating transmitter (Tx) and receiver (Rx) channels of AWR2944; (**b**) spatial relationships of virtual TRx channels.

Figure 2a shows the antenna layout of the AWR2944, comprising multiple transmitter (Tx) and receiver (Rx) channels, with λ/2 spacing between the Rx channels and 2λ spacing between the Tx channels [25]. Although Tx0 was designated for elevation-angle processing, it was excluded from the measurements because this study focuses solely on azimuth-angle data. Instead, Rx1, Rx2, Rx3, and Rx4 receiver channels, along with Tx1, Tx2, and Tx3 transmitter channels, were used for the DDMA-based MIMO configuration.

Figure 2b illustrates the configuration of the TRx channels formed by combining the Tx and Rx channels. Different phase shifts are applied to each Tx channel, enabling spatial separation of the signals from each Tx channel. Each Tx channel pairs with all Rx channels, creating a set of virtual channels to enhance the radar performance. The TRx channels form a virtual uniform linear array with a spacing of λ/2, which enables precise azimuth-angle estimation by utilizing the unique phase shifts and spatial arrangement of the channels.

**Figure 3:**
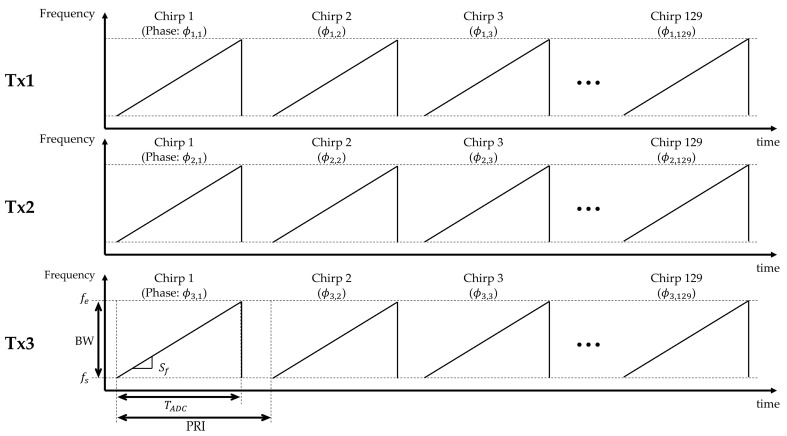
Tx waveform of DDMA-based MIMO radar, showing distinct phase shifts for each transmitter.

Figure 3 shows the Tx waveform structure used in the DDMA-based MIMO radar system. The system employs three Tx channels (Tx1, Tx2, and Tx3) with unique phase shifts ($\phi_{n}$) of 0, 2/3π, and −2/3π, respectively. The phase shift for each chirp ($\phi_{n.k}$) can be represented by the following equation:

(1) $$\phi_{n.k}=\phi_{n}\times (k-1),$$

where *n* denotes the Tx channel and *k* indicates the chirp number.

These Tx channels emit chirp signals within a defined frequency range. The key parameters of the transmitted waveform, including the bandwidth (*BW*), frequency slope ($S_{f}$), ADC sampling time ($T_{ADC}$), and pulse repetition interval (*PRI*), are provided in Table 1.

The waveform employed in this study operated within a frequency range of 77.1–77.31 GHz, with a *BW* of 211.35 MHz, sampling rate (*SR*) of 20 MHz, and $T_{ADC}$ of 12.8 µs. The range resolution ($R_{res}$) was calculated to be 0.71 m according to Equation (2) [26,27]:

(2) $$R_{res}=\frac{c}{2\;BW},$$

where *c* is the speed of light. The maximum detection range ($R_{max}$) was determined to be 90.78 m using Equation (3) [26,27], as follows:

(3) $$R_{max}=\frac{c\cdot T_{ADC}\cdot SR}{4\;BW}\;$$

The wavelength (λ) at the center frequency ($f_{c}$) of 77.2 GHz, within the operational frequency range of 77.1–77.31 GHz, was calculated to be 3.883 mm. The velocity resolution ($V_{res}$) was derived to be 0.38 m/s using Equation (4) [26,27]:

(4) $$V_{res}=\frac{c}{2\;f_{c}\cdot PRI\cdot N_{c}\;}=\frac{\lambda }{2\cdot PRI\cdot N_{c}}\;$$

Lastly, the maximum velocity ($V_{max}$) was calculated to be 24.27 m/s using the following equation [26,27]:

(5) $$V_{max}=\frac{c}{4\;f_{c}\cdot PRI}=\frac{\lambda }{4\cdot PRI}\;$$

#### 2.1.3. Measurement Methods

To ensure reliable measurements in the automotive radar system, antenna calibration data were generated as follows:

(6) $${calibration}_{antenna}=\frac{A_{ch_{1}}}{A_{ch_{n}}}\times e^{-i\left({phase}_{ch_{n}}-{phase}_{ch_{1}}\right)},$$

where $A_{ch_{1}}$ and $A_{ch_{n}}$ represent the amplitudes of the first and *n*-th channels, respectively. Similarly, ${phase}_{ch_{1}}$ and ${phase}_{ch_{n}}$ denote the phases of the first and *n*-th channels.

The calibration data were normalized to channel 1 and applied to the measured data by multiplying each channel accordingly. To ensure the proper alignment for baseline measurements, the radar mounting angle was verified and adjusted to 0° through test drives on public roads.

**Figure 4:**
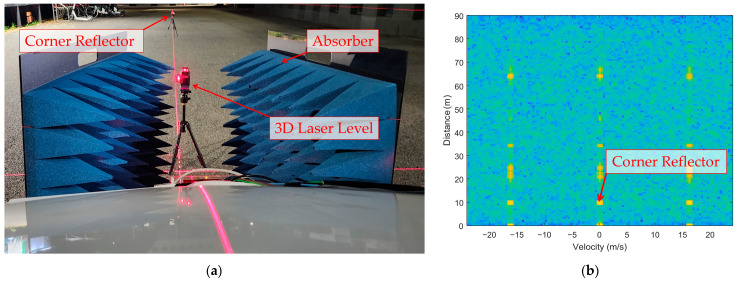
Mounting angle measurement of automotive radar: (**a**) measurement setup; (**b**) resulting range–velocity (RV) spectrum.

Figure 4 presents the setup used to measure the mounting angle of the automotive radar system.

Figure 4a shows a corner reflector precisely positioned at a distance of 10 m and aligned at 0° azimuth relative to the vehicle-mounted radar. This configuration served as the baseline reference for subsequent mounting angle adjustments. To minimize interference from unwanted reflections, radio frequency (RF) absorbers were placed on both sides of the radar, effectively narrowing the field of view (FOV) and isolating the signal from the corner reflector. By physically blocking the left and right sides of the radar, the absorbers ensured a clear focus on the intended target. A 3D laser level was used to establish a baseline reference for systematically introducing mounting angle offsets during subsequent tests. The laser alignment facilitated the precise alignment between the antenna center of the radar and the corner reflector, providing a reliable reference for the measurements. The range–velocity (RV) spectrum shown in Figure 4b validates the setup. A prominent reflection from the corner reflector was observed in the 10 m range, with minimal interference detected beyond this point. The absorbers effectively reduced the noise, ensuring that the radar measurements were concentrated solely on the corner reflector. This setup provided a robust foundation for analyzing the impact of mounting angle adjustments on radar performance.

**Figure 5:**
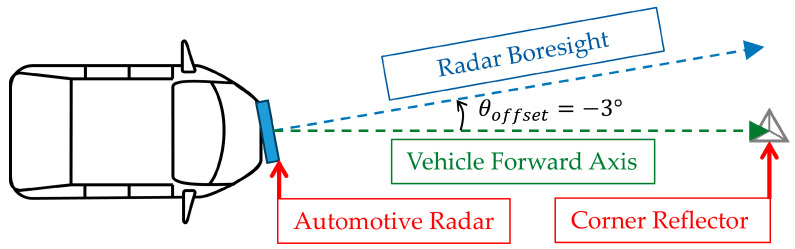
The adjustment of the automotive radar mounting angle to introduce a −3° misalignment offset ($\theta_{offset}$).

**Figure 6:**
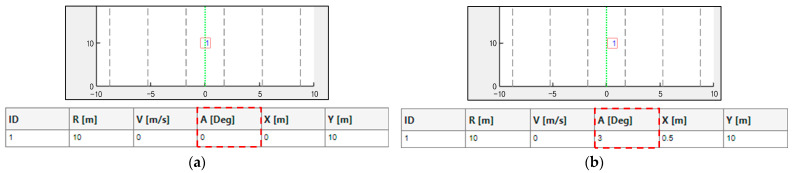
Validation results using in-house developed tool, with corner reflector located at 10 m and 0°: (**a**) radar mounting angle offset: 0°; (**b**) radar mounting angle offset: −3°.

Figure 5 demonstrates the method used to adjust the mounting angle of the automotive radar. Initially, the corner reflector was aligned with the forward axis of the vehicle, corresponding to a mounting angle of 0°, as established using a 3D laser level during the baseline setup. An in-house-developed tool was employed to verify the precise positioning of the corner reflector and to ensure the accurate alignment of the mounting angle of the radar. The corner reflector was then repositioned to −3° relative to the vehicle’s forward axis at the same distance. Subsequently, the mounting angle of the automotive radar was physically adjusted such that the −3° corner reflector was aligned with the radar boresight. This adjustment resulted in the radar being misaligned by −3° ($\theta_{offset}$) relative to the vehicle’s forward axis. After the radar was adjusted to this offset, the corner reflector was returned to the baseline position marked using the 3D laser level to verify the offset alignment. Under these conditions, the automotive radar detected the corner reflector at +3°, reflecting the effect of the intentional mounting angle offset ($\theta_{offset}$). The verification of this setup and the alignment process is illustrated in Figure 6, which shows the output of the in-house-developed tool. In Figure 6a, the radar mounting angle offset is 0°, and the corner reflector is detected at 10 m and 0°. As shown in Figure 6b, after a −3° mounting angle offset was introduced to the radar, the corner reflector at the same position was detected at +3°.

With this approach, mounting angle offsets of −3°, −2°, −1°, 0°, +1°, +2°, and +3° were systematically introduced. Driving data were then collected for each offset configuration to create a dataset of radar measurements under set mounting angle offsets.

### 2.2. Preprocessing

#### 2.2.1. Structure of Measurement Data

As indicated earlier, the measurement data used in this study were obtained using a DDMA-based MIMO radar system. This section describes the structure of the data collected from this radar, including details on how the DDMA-based approach influences the data configuration.

**Figure 7:**
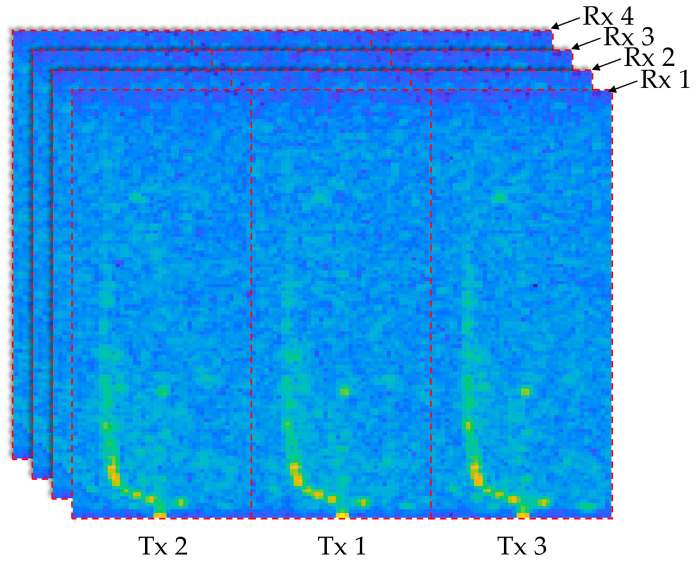
Range–velocity spectrum of measurement data from DDMA-based MIMO radar.

The raw data, acquired from ADC measurements, are initially represented in the time domain and subsequently transformed into the frequency domain using 2D fast Fourier transform (2D FFT). This transformation results in a complex-valued dataset containing both phase and magnitude information. Specifically, applying the 2D FFT yields an RV spectrum with dimensions of 128 (range) by 129 (velocity) for each of the four Rx channels, as shown in Figure 7. The four RV spectra also include signals from three Tx channels, which are separated based on the phase shifts previously described in Section 2.1.2 (0, 2/3π, and −2/3π). The separation of each Tx channel is characterized by a Doppler-related index shift ($i_{shift}$), which indicates the relative position shift along the Doppler axis due to the phase differences applied to the transmit channels per chirp. The value of $i_{shift}$ can be calculated using the following equation:

(7) $$i_{shift}=\frac{∆f_{d}}{∆f_{res}},$$

where $∆f_{d}$ represents the Doppler frequency difference that results from the phase shifts applied to each Tx channel, effectively causing each signal to appear at a distinct position along the Doppler axis, whereas ${∆f}_{res}$ denotes the frequency resolution in the Doppler FFT domain. These values are given by

(8) $$∆f_{d}=\frac{\phi }{2\pi }\times PRF,$$

(9) $$∆f_{res}=\frac{PRF}{N_{c}},$$

where ϕ is the specific phase shift applied to each of the transmit channels (as previously defined in Section 2.1.2); *PRF* is the pulse repetition frequency, which is the reciprocal of the *PRI*; and $N_{c}$ is the number of chirps per frame. These values are then substituted into the equation for $i_{shift}$:

(10) $$i_{shift}=\frac{\phi }{2\pi }\times N_{c}\;$$

Thus, the index shift ($i_{shift}$) depends on the phase shift (ϕ) and the number of chirps ($N_{c}$). Given the phase shift, $i_{shift}$ indicates the extent to which each Tx channel component is shifted along the Doppler axis, allowing for the separation of signals from different Tx channels. This separation enables the independent extraction of velocity information for each Tx channel, ensuring that each transmitted signal can be clearly analyzed in the final RV spectrum.

#### 2.2.2. External Data-Based Preprocessing

In this section, we introduce the filtering process used to create the refined dataset necessary for training. Specifically, we focus on selecting data collected during straight-lane driving and ensuring that a sufficient number of stationary objects are included. To achieve this, the *ego speed* and *heading angle*, which are crucial filtering conditions, were obtained using GPS data, as described in Section 2.1.1. The filtering conditions, including for the constant false-alarm rate (CFAR) level, applied to the data were as follows:

(11) Ego speed ≥10 m/s,

(12) Heading angle deviation≤0.5°,

(13) Ratio of power exceeding CFAR level≥1%.

The *ego speed* threshold of 10 m/s and the *heading angle* deviation threshold of 0.5° were set to ensure straight-line driving. Higher threshold values generally lead to greater driving stability and reduced heading deviations, but they also limit the amount of usable data due to shorter observation periods. These thresholds achieve a balance between capturing reliable measurements and maintaining sufficient sample diversity for effective model training.

An accurate *ego speed* is essential for ensuring the reliability of the dataset. In this study, GPS data were used to collect accurate ego-speed information. However, when GPS data were unavailable, ego-speed correction methods from past studies [28,29,30] could be utilized. The relationship between the *ego speed* and a stationary object is expressed by the following equation:

(14) $$-\frac{V_{r}}{V_{h}}=\cos\left(\theta -\theta_{offset}\right),$$

where $V_{r}$ represents the relative velocity of the stationary target, $V_{h}$ is the *ego speed*, θ is the detection angle of the object, and $\theta_{offset}$ represents the mounting angle offset. A negative sign is included in Equation (14) because the object is stationary, indicating that the relative velocity is in the direction opposite to that of the *ego speed*. An accurate *ego speed* is crucial for estimating the mounting angle offset. Inaccuracies in the *ego speed* can lead to an incorrect estimation of $\theta_{offset}$, thereby affecting the overall reliability of the dataset.

The *heading angle* was determined by analyzing the maximum deviation across frames from −10 to +10 relative to the current frame. Only the data that met the deviation conditions described in Equation (12) were selected. Although GPS data were used to obtain the *heading angle* in this study, the yaw-rate sensor of the vehicle could also be utilized as an alternative for estimating the *heading angle*. To minimize the impact of vehicle maneuvers such as turning, which could introduce unwanted variations, straight-lane driving data were chosen to ensure consistency.

**Figure 8:**
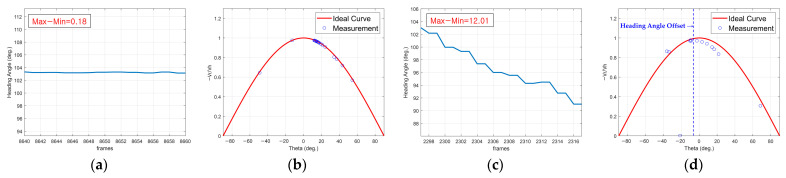
Comparison of cosine curves based on heading angle: (**a**,**b**) heading angle and cosine curve in a straight line; (**c**,**d**) heading angle and cosine curve in a curved lane.

Figure 8 shows a comparison of the cosine curves from Equation (14) for a mounting angle offset of 0°, under different *heading angles*. Figure 8a,b represent straight-lane driving, whereas (c) and (d) represent curved-lane driving. Under straight-lane driving conditions, as shown in (a) and (b), the measured values closely match the ideal cosine curve, indicating minimal deviation. However, significant deviations occur during curved-lane driving, as illustrated in (c) and (d), leading to an offset in the cosine curve. Owing to these deviations, data from curved-lane driving were excluded from the final dataset to avoid inconsistencies and ensure reliable training data.

The CFAR level was set by calculating the median value of each frame to estimate the noise level, followed by applying a 15 dB offset. Frames, where the ratio of power exceeding the CFAR level was below the 1% threshold, were excluded, ensuring that only frames with sufficient power from stationary target detections were retained. This threshold value was determined empirically, based on trials and the characteristics of the radar system, to ensure a sufficient number of stationary objects for training purposes.

#### 2.2.3. TRx Channel Preprocessing

In Section 2.2.2, the dataset was refined through preprocessing steps utilizing external data. The next step involved transforming this dataset into a format suitable for model training.

When the vehicle is stationary, the index of the stationary object for each Rx channel in the RV spectrum can be obtained using Equation (10). This allows segmentation into three distinct channels that contain the stationary object. Extending this segmentation process to all four Rx channels yields a total of twelve channels. However, Equation (10) is applicable only in the stationary-vehicle scenario (*ego speed* = 0), because it is used to determine the mounting angle offset of the radar with stationary objects. Therefore, for effective training, it is necessary to determine the index of the stationary object even when the vehicle is in motion (*ego speed* > 0). To accommodate both stationary- and moving-vehicle conditions, the index of the stationary object can be represented as follows:

(15) $$I_{shift}=i_{shift}-i_{ego},$$

where $I_{shift}$ denotes the final index of the stationary object after all corrections are applied; $i_{shift}$ is the initial index of the stationary object, given by Equation (10); and $i_{ego}$ is the index offset caused by the *ego speed* of the vehicle, accounting for the movement of the vehicle itself. The negative sign accounts for the effect of the *ego speed*, which causes stationary objects to shift in the opposite direction in the Doppler spectrum. The index offset $i_{ego}$ can be defined as

(16) $$i_{ego}=\frac{ego\;speed}{V_{res}},$$

where $V_{res}$ denotes the velocity resolution, as defined in Table 1. Each Doppler index corresponds to a velocity increment of $V_{res}$; thus, dividing the *ego speed* by $V_{res}$ provides the associated Doppler index offset.

Equation (15) effectively adjusts the index to compensate for the *ego speed*, ensuring an accurate representation of stationary objects in the RV spectrum for model training. With this adjusted index from Equation (15), the stationary object for each TRx channel was aligned to the same index, resulting in 12 separate data segments, each with dimensions of 128 (range) × 43 (velocity), as shown in Figure 7. Subsequently, antenna calibration values corresponding to each channel were applied.

Up to now, the data generated contained ego-speed information from Equation (16). Therefore, it is highly likely that this latent ego-speed information will be incorporated during model training. To validate this, an additional channel was created as a 128 × 43 matrix composed entirely of ego-speed data.

**Figure 9:**
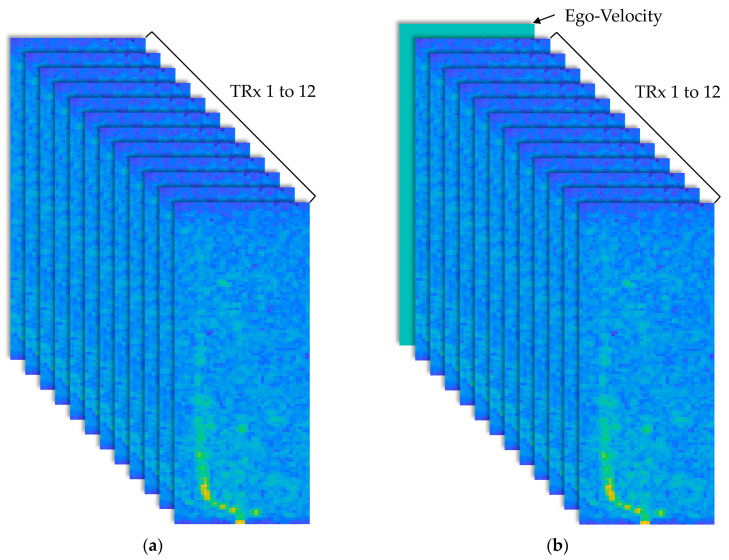
Data structure prepared for CV-CNN model input: (**a**) without additional ego-speed channel; (**b**) with additional ego-speed channel.

Figure 9 shows the structure of the data used as input for the CV-CNN model. The data with antenna calibration applied were then structured as 128 (range) × 43 (velocity) × 12 (TRx channels) per frame, to be used as an input for the training model, as shown in Figure 9a. Figure 9b shows the addition of an ego-speed channel to verify whether the latent ego-speed information had been effectively integrated.

Through these preprocessing steps, a total of 36,175 frames were processed, as summarized in Table 2. The total number of frames resulting from preprocessing is shown alongside the mounting angle offsets, ranging from −3° to +3°. These frames were further split into training (80%), validation (10%), and test (10%) datasets.

**Figure 10:**
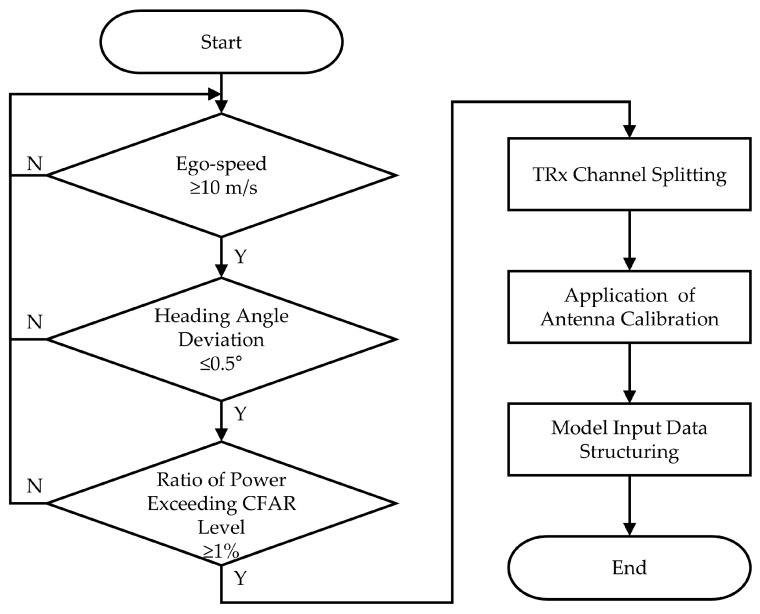
Flowchart of overall preprocessing.

Figure 10 summarizes the overall preprocessing described in Section 2.2.1, Section 2.2.2 and Section 2.2.3. The process begins with external data-based preprocessing, including ego-speed filtering, heading-angle deviation checks, and CFAR level thresholding. Once these conditions are satisfied, the data are split into TRx channels, antenna calibration is performed, and the data are structured for input into the CV-CNN model.

### 2.3. Design of CV-CNN Model

#### 2.3.1. Overview of CV-CNN

Radar system data are primarily represented in complex form. Complex-valued data contains both amplitude and phase information, which conveys essential details about the distance, speed, and reflective properties of objects. Typically, the amplitude represents the strength of the reflected signal, while the phase corresponds to the time delay and frequency shifts associated with changes in the object’s position or velocity.

While conventional CNNs are designed to learn and extract features from real-valued data, CV-CNNs are specifically designed to process complex-valued inputs directly. This is achieved through the implementation of complex convolutions, enabling the network to effectively capture intricate variations in both the phase and amplitude present in radar data. Such an approach enhances the model’s ability to leverage the complex-valued characteristics of radar signals, allowing for more precise learning of subtle variations in phase and amplitude. The complex convolution operation in CV-CNN is performed as follows:

(17) $$z=\left(a+ib\right)\times \left(w+iv\right),$$

where a and b represent the real and imaginary components of the input data, and w and v denote the real and imaginary parts of the kernel weights. This CV-CNN approach is applied to the AutoRAD-Net model, facilitating the effective learning of mounting angle offsets from the radar data.

#### 2.3.2. Architecture of AutoRAD-Net Model

The proposed AutoRAD-Net model, based on a CV-CNN, was designed for estimating mounting angle offsets in automotive radar systems. As described in Section 2.2.3, two models were constructed to evaluate whether latent ego-speed information had been effectively integrated. AutoRAD-Net utilizes 12 preprocessed TRx channels for the input, whereas AutoRADs-Net incorporates an additional ego-speed channel, resulting in 13 input channels. Table 3 summarizes the input–output structures of each layer for both models.

**Figure 11:**
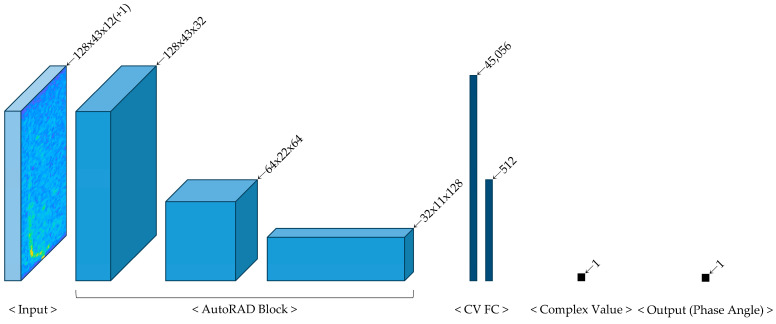
Architecture of AutoRAD-Net.

**Figure 12:**
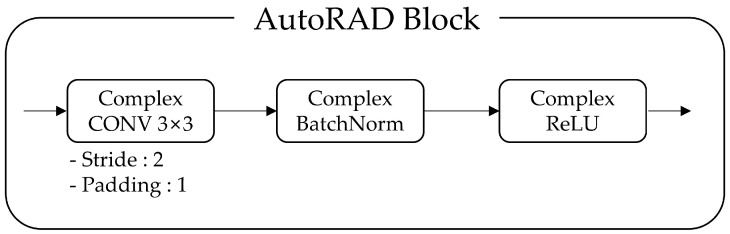
Configuration of AutoRAD-Net.

The overall architecture of the proposed models is illustrated in Figure 11, providing a detailed visualization of the network flow from input to output. Both models follow a common structure consisting of three complex-valued convolutional (CV CONV) layers, collectively referred to as the AutoRAD Block, and two complex-valued fully connected (CV FC) layers. The input data are represented as 128 × 43 × 12 (+1), where 12 corresponds to the number of preprocessed TRx channels in AutoRAD-Net. The additional +1 channel, representing ego-speed information, is included exclusively in AutoRADs-Net, making a 13-channel input. Within the AutoRAD Block, each layer extracts features and progressively transforms the data into intermediate representations with dimensions of 128 × 43 × 32 and 64 × 22 × 64. Each AutoRAD Block consists of a complex convolutional layer, complex batch normalization, and a complex rectified linear unit (ReLU) activation function. The complex convolutional layer is configured with a 3 × 3 kernel, stride of 2, and padding of 1, as shown in Figure 12. This design efficiently processes radar FFT data and extracts meaningful patterns and complex-valued information that are critical for estimating the mounting angle offset. To process these extracted features efficiently, two CV-FC layers are employed [31]. The final processed features yield complex-valued outputs, which are used to calculate the phase angle for estimating the mounting angle offset.

The model was trained using the adaptive moment estimation optimizer with a learning rate of 0.001 and a batch size of 64. For the loss function, the mean squared error (MSE) was used to minimize the difference between the predicted and actual mounting angle offsets. MSE was chosen because it effectively increases the loss for larger deviations by squaring the error, making it particularly suitable for continuous regression tasks where minimizing large errors is essential. These selected hyperparameters, including the optimizer, learning rate, and loss function, work together to ensure efficient model training and contribute to optimizing mounting angle offset estimation for automotive radars.

## 3. Results and Discussion

**Figure 13:**
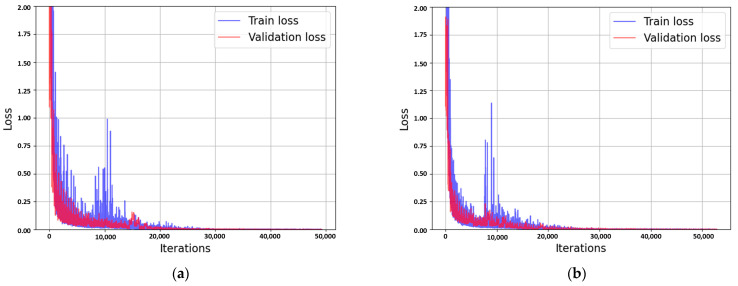
Training and validation loss: (**a**) AutoRAD-Net; (**b**) AutoRADs-Net.

As shown in Figure 13, both the AutoRAD-Net and AutoRADs-Net models achieved a validation loss close to zero, indicating that both models effectively captured the key features in the training data. Notably, the AutoRAD-Net model, which did not include an ego-speed channel, also achieved a validation loss of nearly zero. This provides evidence supporting the hypothesis that ego-speed information has already been implicitly incorporated through the process described in Equation (16), as explained in Section 2.2.3. These results suggest that the inclusion of an additional ego-speed channel may not be necessary. The AutoRAD-Net model was able to learn the relevant features effectively without it, similar to AutoRADs-Net, which explicitly included an ego-speed channel. The implicit incorporation of ego-speed information through Equation (16) appears to provide sufficient information for the model to perform well, rendering the explicit inclusion of the ego-speed channel redundant.

However, although a validation loss approaching zero suggests effective learning on the validation dataset, it is important to recognize that this result is specific to the dataset used during training. Therefore, it is essential to evaluate the performance of the models on a separate unseen test dataset to ensure their generalizability.

**Figure 14:**
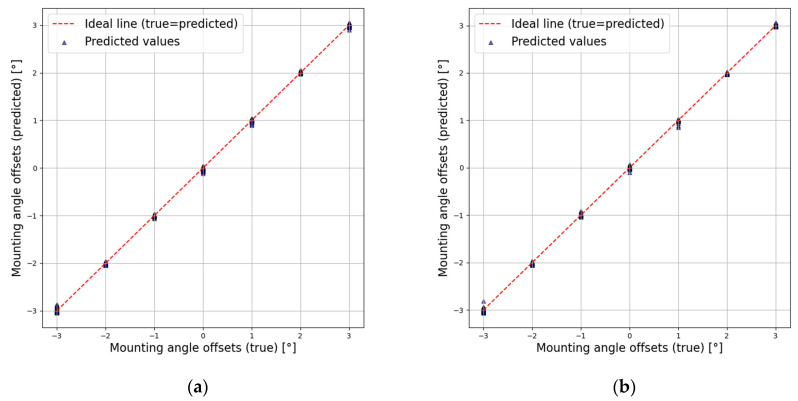
True and predicted mounting angle offsets: (**a**) AutoRAD-Net; (**b**) AutoRADs-Net.

Figure 14 presents the test results for both the AutoRAD-Net and AutoRADs-Net models on the test dataset. The red dashed line represents the ideal, where the actual mounting offset values align with the model predictions, whereas the triangles provide scatter plots of the angles predicted by the models. Both models accurately estimated the mounting offset of the automotive radar, with an error margin of 0.2°. This further supports the earlier explanation that the AutoRAD-Net model, which does not rely on additional ego-speed channel information, was able to accurately estimate the radar offset. This also reinforces the inference that the inclusion of explicit ego-speed information is unnecessary, given that potential ego-speed information is already implicitly incorporated.

**Figure 15:**
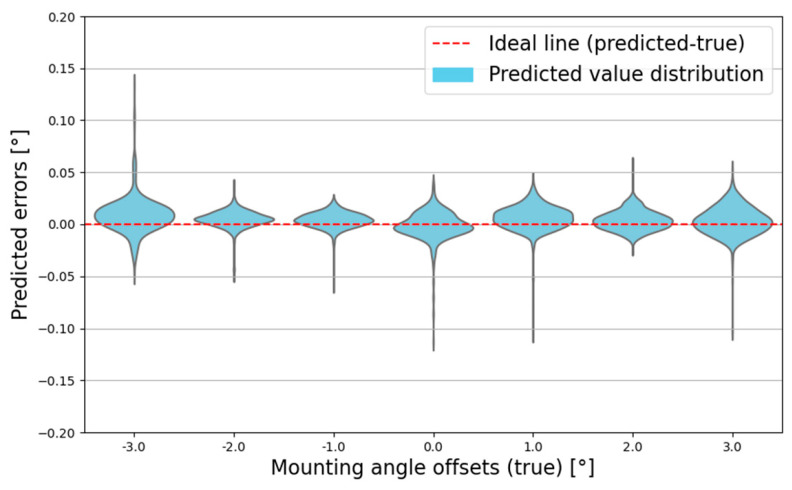
Predicted-angle errors on test dataset using AutoRAD-Net.

Figure 15 presents a violin plot that visualizes the distribution and density of the differences between the predicted and actual mounting offsets for the AutoRAD-Net model. The red dashed line indicates the ideal alignment, where the predictions perfectly match the actual values. The plot shows that the errors range within ±0.15°, with the highest-density region closely aligned with the ideal line. This highlights the high accuracy and consistent reliability of the model across all tested mounting offsets.

However, although the model was trained with specific mounting offsets, the goal of this study was to design a regression model capable of predicting arbitrary mounting offsets. To test this, we acquired data for a mounting offset of −1.7° and processed the data according to the preprocessing steps described in Section 2.2. Additionally, we compared the model’s performance with conventional methods by estimating mounting angle offsets from a single frame using Equation (14) [10,11,12].

**Figure 16:**
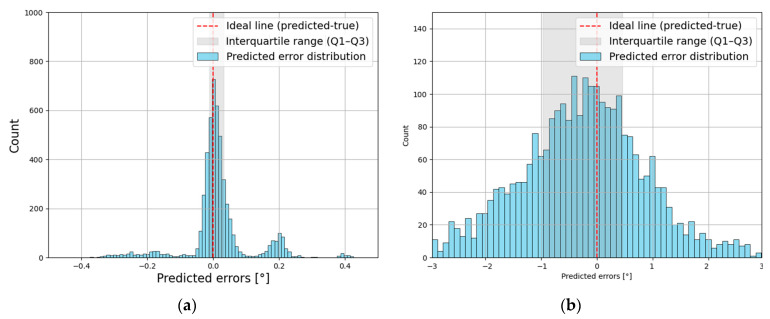
Predicted-angle error distribution at a mounting angle offset of −1.7°: (**a**) AutoRAD-Net; (**b**) conventional method.

Figure 16 presents the prediction results of the AutoRAD-Net model for a mounting offset of −1.7°, which differs from the mounting offsets used in the training dataset (comprising offsets of −3°, −2°, −1°, 0°, +1°, +2°, and +3°). The figure also presents the corresponding results obtained using the conventional method. The blue boxes in the figure show the histogram distribution of the error (i.e., the difference between the predicted and actual mounting angle offsets), whereas the red dashed line represents the ideal, where the predicted mounting angle offset perfectly aligns with the actual value. Meanwhile, the gray shaded area indicates the interquartile range (*Q*1–*Q*3), which is the range between the 25th percentile (*Q*1) and 75th percentile (*Q*3) of the error distribution. This range highlights the middle 50% of the error values, providing a clear visualization of the concentration of prediction errors around the true mounting angle offset. Table 4 lists the mounting angle prediction errors derived from the data shown in Figure 16. This table summarizes the key statistical measures of the prediction errors and provides a more detailed numerical analysis of the results illustrated in the figure.

The median error for AutoRAD-Net is exceptionally small, at $6.97\times 10^{-3}$°, indicating that the predictions of the model were generally very close to the true angle. In contrast, the conventional method exhibits a significantly larger median error of $-213.30\times 10^{-3}$°. While the minimum and maximum errors for AutoRAD-Net are $-373.51\times 10^{-3}$° and $424.14\times 10^{-3}$°, respectively, these values are substantially smaller than those of the conventional method, which are $-3258.67\times 10^{-3}$° and $3953.98\times 10^{-3}$°. Furthermore, the interquartile range (IQR) for AutoRAD-Net, defined by the 25th percentile ($Q1=-11.97\times 10^{-3}$°) and the 75th percentile ($Q3=32.20\times 10^{-3}$°), demonstrates that the majority of prediction errors are concentrated within a narrow range. By contrast, the IQR of the conventional method is significantly wider, with *Q*1 at $-973.69\times 10^{-3}$° and *Q*3 at $457.34\times 10^{-3}$°. These findings confirm that AutoRAD-Net delivers highly reliable predictions even for a mounting offset not included in the training dataset, outperforming the conventional method, which exhibits greater variability and larger errors.

## 4. Conclusions

In this study, we introduced the AutoRAD-Net model, developed using CV-CNN architecture to address the challenge of automotive radar alignment, specifically targeting azimuth misalignment. The model demonstrated high accuracy in estimating mounting angle offsets. The training and validation datasets included mounting angle offsets of −3°, −2°, −1°, 0°, +1°, +2°, and +3°. When tested on these offsets, the model achieved prediction errors not exceeding 0.15°. Additionally, the model was evaluated on a new mounting offset of −1.7°, which was not included in the training dataset. While the performance of the model on the −1.7° offset was slightly lower than that on the training offsets, the error distribution confirmed its ability to make reliable predictions. When compared to the conventional method on the same −1.7° offset, AutoRAD-Net exhibited significantly better performance, achieving smaller error margins and more consistent predictions. These results demonstrate the robustness of the AutoRAD-Net model in handling unseen mounting angle offsets and its adaptability to new data.

The predicted mounting angle offsets can be utilized in a practical manner in two ways. First, they can be used to physically adjust the mounting angle of the radar to ensure optimal alignment. Second, the predicted offsets can be applied in radar systems using compensation algorithms, where the mounting angle offset is compensated by the angle information of the object. This allows for more accurate sensor data interpretation, especially in safety and convenience systems such as collision avoidance and ACC. By incorporating this approach, automotive radar systems can achieve more precise alignment, improving the reliability and performance of radar sensors, particularly in autonomous driving applications.

However, although the AutoRAD-Net model demonstrated strong performance in estimating mounting angle offsets, there were certain limitations to this study. One limitation was that the model was trained with a limited range of mounting angle offsets, specifically −3°, −2°, −1°, 0°, +1°, +2°, and +3°. The ability of the model to generalize extreme or rare mounting offsets outside this range requires further validation. Additionally, although the model provided accurate predictions for the −1.7° offset, its performance was slightly lower compared to that of the training offsets, indicating that further improvements may be needed to handle data points significantly deviating from the training set. Future studies could address these limitations by incorporating more diverse training data and enhancing the robustness of the model to accommodate a wider range of mounting offsets. Furthermore, integrating AutoRAD-Net with existing research approaches [10,11,12], which have primarily focused on post-processing techniques such as filtering or statistical methods across multiple data frames, has the potential to further enhance angle estimation performance, providing a more comprehensive solution for automotive radar alignment.

## Figures and Tables

**Table 1:** Waveform specifications for DDMA MIMO radar system.

Parameter	Value	Parameter	Value
$\mathrm{Start}\;\mathrm{frequency}\;(f_{s}$)	77.10 GHz	$\mathrm{Number}\;\mathrm{of}\;\mathrm{ADC}\;\mathrm{samples}\;(N_{s}$)	256
$\mathrm{End}\;\mathrm{frequency}\;(f_{e}$)	77.31 GHz	$\mathrm{Number}\;\mathrm{of}\;\mathrm{chirps}\;(N_{c}$)	129
Bandwidth (*BW*)	211.35 MHz	$\mathrm{Frame}\;\mathrm{duration}\;(T_{frm}$)	50 ms
Sample rate (*SR*)	20 MHz	$\mathrm{Range}\;\mathrm{resolution}\;(R_{res}$)	0.71 m
$\mathrm{ADC}\;\mathrm{sampling}\;\mathrm{time}\;(T_{ADC}$)	12.8 µs	$\mathrm{Maximum}\;\mathrm{range}\;(R_{max}$)	90.78 m
$\mathrm{Frequency}\;\mathrm{slope}\;(S_{f}$)	16.51 MHz/µs	$\mathrm{Velocity}\;\mathrm{resolution}\;(V_{res}$)	0.38 m/s
Pulse repetition interval (*PRI*)	40 µs	$\mathrm{Maximum}\;\mathrm{velocity}\;(V_{max}$)	24.27 m/s

**Table 2:** Datasets after preprocessing.

Mounting Angle Offsets	Preprocessed Frames (100%)	Split Frames		
		Training (80%)	Validation (10%)	Test (10%)
−3°	5454	4363	545	546
−2°	5118	4094	512	512
−1°	5110	4088	511	511
−0°	5661	4528	566	567
+1°	5197	4157	520	520
+2°	4895	3916	489	490
+3°	4740	3792	474	474
Total frames	36,175	28,938	3617	3620

**Table 3:** Input and output structures of each layer in the Automotive Radar Alignment Detection Network (AutoRAD-Net) model.

Layer Name	Input Size	Output Size	AutoRAD-Net		AutoRADs-Net	
			Channels		Channels	
			In	Out	In	Out
Input	-	128 × 43	-	12	-	13
CV CONV 1	128 × 43	128 × 43	12	32	13	32
CV CONV 2	128 × 43	64 × 22	32	64	32	64
CV CONV 3	64 × 22	32 × 11	64	128	64	128
CV FC1	45,056	512	-	-	-	-
CV FC2	512	1	-	-	-	-
Parameters	-		46,331,522		46,332,098	

**Table 4:** Comparison of mounting angle prediction errors for a true offset of −1.7° using AutoRAD-Net and the conventional method.

Category	AutoRAD-Net	Conventional Method
Median angle	$6.97\times 10^{-3}$°	$-213.30\times 10^{-3}$°
Minimum angle	$-373.51\times 10^{-3}$°	$-3258.67\times 10^{-3}$°
Maximum angle	$424.14\times 10^{-3}$°	$3953.98\times 10^{-3}$°
25th percentile (*Q*1)	$-11.97\times 10^{-3}$°	$-973.69\times 10^{-3}$°
75th percentile (*Q*3)	$32.20\times 10^{-3}$°	$457.34\times 10^{-3}$°

## Data Availability

The datasets presented in this article are not readily available due to proprietary concerns. Requests to access the datasets should be directed to the corresponding author.
